# Honey bee death from aerosols inadvertently produced from propelled aerial dispersal of a solid ant bait

**DOI:** 10.1002/ps.7140

**Published:** 2022-09-26

**Authors:** Benjamin D. Hoffmann, Greg Quinn

**Affiliations:** ^1^ CSIRO Health & Biosecurity, Tropical Ecosystems Research Centre Winnellie Northwest Territories Australia; ^2^ Aerospread Technologies Limited Napier New Zealand

**Keywords:** *Apis mellifera*, fipronil, hydrogel, impacts, invasive, nontarget impacts

## Abstract

**Background:**

Hydrogels are a new bait form, and multiple studies have found minimal potential or actual nontarget impacts associated with their use. However, in 2020, aerial applications of hydrogels containing fipronil unequivocally resulted in honey bee deaths. Here we detail four studies that then were conducted to determine how the bees were exposed to the active constituent and how to modify the aerial treatment protocols to eliminate the risk to bees.

**Results:**

The first trial confirmed the existence of fipronil in aerosol form. The second trial quantified that in specific wind conditions the aerosols were falling to the ground at a maximum distance determined by an approximate 30° angle from the ground to the dispersal point, and that free‐falling hydrogels either do not produce aerosols or, if so, at volumes too negligible to be collected or quantified. The third trial confirmed that even bee hives upwind and several hundreds of metres away from the area being baited can be contaminated by the aerosols. The fourth trial found no bee hive mortality as a result of either free‐falling bait or moving bee hives 500 m beyond the treatment area.

**Conclusion:**

The aerosol issue is likely to occur with every motorized mechanism dispersing hydrogels. It is possible that the same issue happens with solid dry products if they produce a fine dust when propelled during dispersal. Further research into this issue is warranted. © 2022 The Authors. *Pest Management Science* published by John Wiley & Sons Ltd on behalf of Society of Chemical Industry.

## Graphical abstract

Aerial applications of hydrogels containing fipronil unequivocally resulted in honey bee deaths. We confirmed that aerosols were being produced when the product was propelled for dispersal. It is possible that the same issue happens with solid dry products if they produce a fine dust when propelled during dispersal.

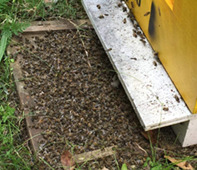

## 1 INTRODUCTION

The minimization or mitigation of nontarget impacts is a major focus of invasive species management programs.^1^, ^2^ In some circumstances merely changing the treatment product or device design will mitigate such impacts,^3^, ^4^ but in other circumstances mitigation can involve changes in practice such as placing baits in bait stations,^5^ utilizing baits that are as species‐specific as possible,^6^ or even greater efforts such as encouraging or physically removing nontarget species from treatment areas.^7^, ^8^, ^9^

Solid ant baits can be dispersed by many methods,^10^, ^11^, ^12^ with aerial dispersal of bait being commonly used when areas are too large or difficult for the work to be conducted using ground‐based dispersal methods. Aerial dispersal of solid ant baits typically involves the bait not being free‐fall dropped but instead being propelled out by some means to maximize the swath distance (spread), thereby reducing the number of flight paths needed. The use of a propelling device is a standard delivery mode that also is used for the likes of aerial dispersal of plant seeds, fertilizers and rodent bait.^13^, ^14^, ^15^

In 2012, the novel bait form of hydrogels (superabsorbent polymers that can absorb liquids) was used for the first time against ants.^16^ The use of hydrogels for ant control was ingenious in that it provided a liquid food substance that can be imbibed by ants,^17^ but in a solid form which allows for ground or aerial dispersal. To make such a bait is easy; hydrogels just absorb a sucrose solution containing an active constituent that affects the ants. The use of hydrogels has proven to be highly efficacious against multiple ant species in broadscale applications,^18^, ^19^, ^20^ including on Norfolk Island (29° 02′  S, 167° 57′  E) in the Pacific Ocean, where hydrogels have been used since 2016 as a primary treatment product within an Argentine ant *Linepithema humile* eradication program, and have been dispersed there aerially since 2018.

**Figure 1:**
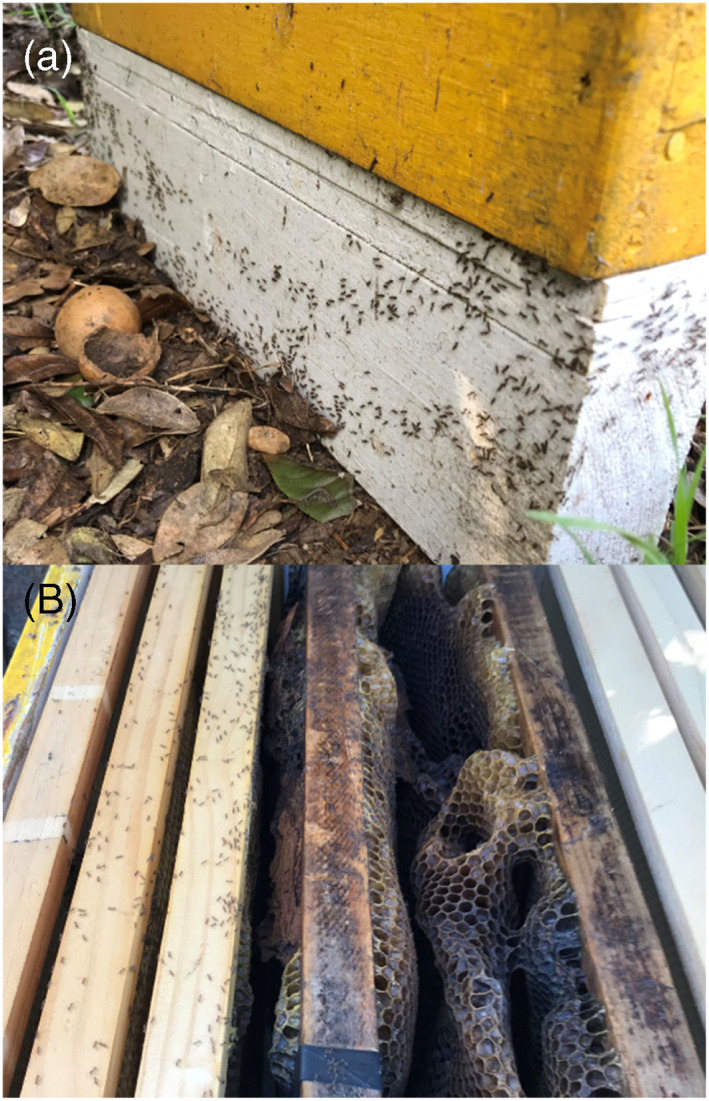
(a) Argentine ants with a large recruitment trail to a living bee hive, and (b) Argentine ants consuming the honeycomb from a hive that they recently killed.

Hydrogels have been used extensively on Norfolk Island in numerous locations, accompanied by multiple studies investigating the potential for nontarget impacts (Hoffmann in press). Specifically it has been found that honey bees (*Apis mellifera*; hereafter referred to as bees) are not attracted to hydrogels, hydrogels ground‐dispersed around bee hives do not generate hive collapse, and nontarget impacts of soil invertebrates are negligible such that seasonal variation and impacts of Argentine ant are greater than any short‐term and localized impacts resulting from consumption of the bait. The lack of attractancy and impacts on bees also has been found in other studies elsewhere.^21^, ^22^ For these reasons the aerial distribution of hydrogels was deemed to have no inherently greater risk for any nontarget species than ground‐based dispersal, especially bees. By contrast, bee hives (Fig. 1) and many other fauna are readily killed by Argentine ants.^23^, ^24^

In 2020, aerial treatments using hydrogels unequivocally resulted in bee deaths, but at the time it was not completely clear how. Here we detail the research conducted to determine as far as practicable how the bees were contaminated by the treatments, how we modified the aerial treatment protocols to avoid future nontarget impacts, and additional lessons that were learned in subsequent aerial treatments that further modified our aerial treatment protocols that have not since resulted in bee deaths.

## 2 MATERIALS AND METHODS

### 2.1 Hydrogel ant bait and aerial dispersal

Hydrogel ant bait for all work was prepared by placing hydrogels in 30% sugar‐water solutions containing 0.006 mL L^–1^ fipronil [sourced from Termidor® (BASF, Florham Park, NJ, USA) containing 100 g L^–1^ fipronil] (i.e. a mixture of water:sugar:Termidor® of 350:150:0.003), and allowing 24 h for the solution to be absorbed. Sugar used was standard 1A sugar from the New Zealand Sugar Co. Ltd, Aukland, New Zealand), and the hydrogels were Water$ave Floragel® from Polymer Innovations Pty Ltd, Singleton, NSA, Australia). The bait was prepared 24 h before use in all instances.

The bait dispersal mechanism was a 300‐mm granular spreading disk, hereafter referred to as the spinner, spinning at 720 rpm which propelled the hydrogels out with centrifugal force as they flowed down from a holding container, in the same manner as any manual fertilizer spreader.

### 2.2 Background

**Figure 2:**
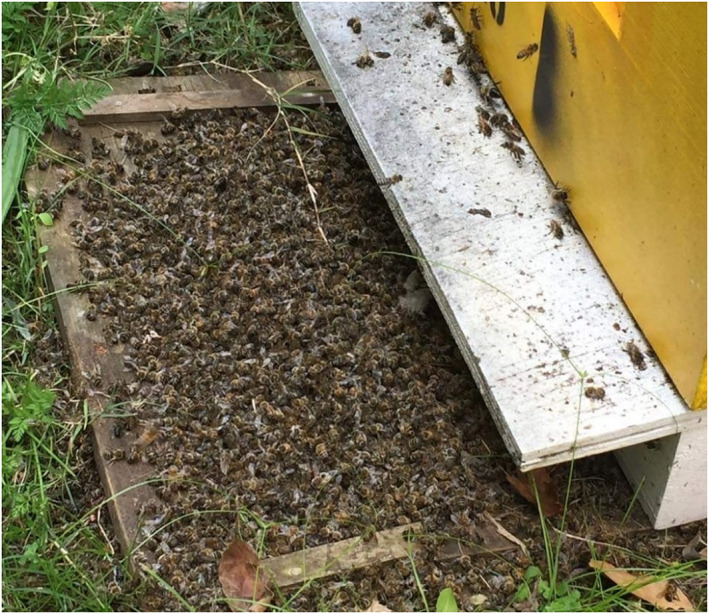
Dead and dying bees at the entrance of one of the affected bee hives on Norfolk Island.

**Figure 3:**
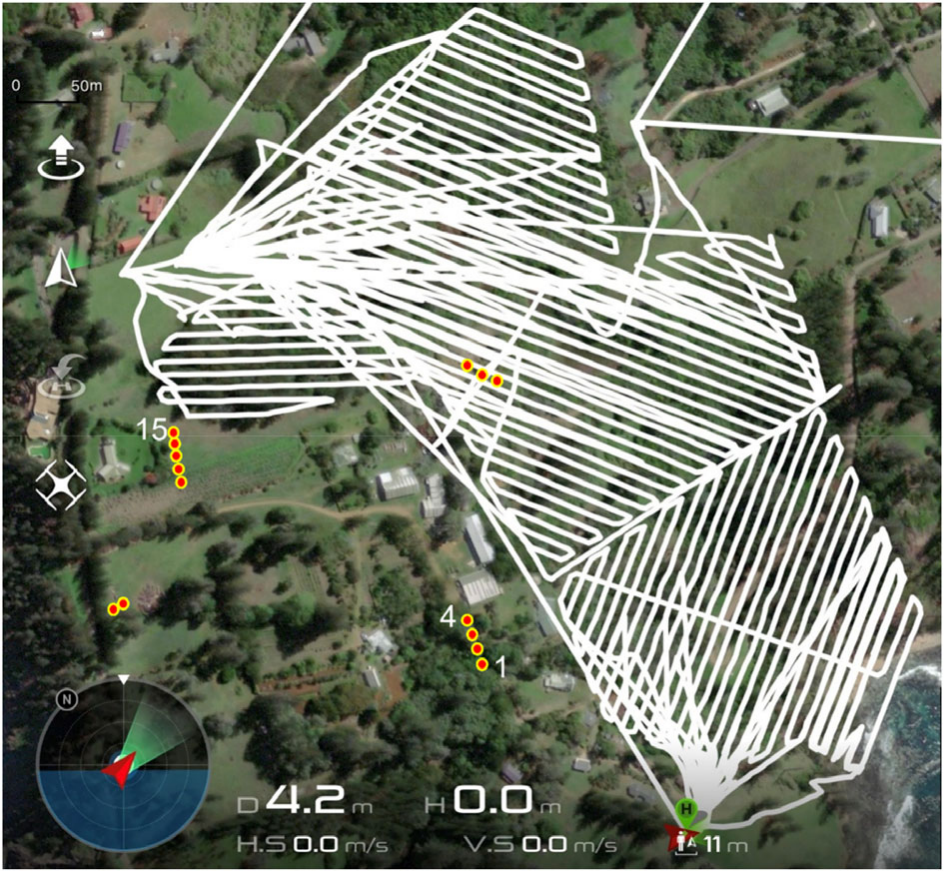
On‐ground situation at the time of the bee kill, showing the flight paths of the second treatment (white lines) conducted on 1 and 2 October 2020, and locations of the 15 bee hives (red circles) in the immediate vicinity. Numbers 1, 4 and 15 are the bee hives from where dead bees were collected and sent for analysis for the presence of fipronil. All other symbols are from the screen display of the aerial delivery computer system. Wind was 16 knots (5 m s^–1^) easterly.

The day after the second aerial treatment was completed, one week after the first treatment was conducted, project staff were alerted to high numbers of dead and dying bees at the entrance of commercial honey bee hives in close proximity (0–200 m) and downwind of the treatment area (Figs 2 and 3). Before this time the managers of the bee hives were observing no dead bees and very high foraging activity from the hives in line with expected bee activity levels in spring. Treatments ceased immediately, and handful‐sized volumes of dead bees were collected from three hives, placed in plastic bags, and posted to the Australian National Measurement Institute (NMI) for assessment of fipronil presence using gas chromatography with mass spectrometry (GC–MS). For this assessment, a 1 g representative portion of each sample was extracted by soaking overnight in 10 mL acetonitrile. Each sample then was shaken on horizontal shaker for 5 min before being centrifuged at 3500 rpm for 5 min at room temperature. An aliquot of each sample extract was then transferred to a GC vial. Analyte protectants were added for quality assurance and performance purposes, and the extract was then analzsed using GC–MS. This test had a sensitivity of 0.01 mg kg^–1^.

The following day bees continued to be found dead at the entrances of these hives, but in lower numbers. It also was confirmed that government sentinel hives located around the island for biosecurity monitoring purposes did not have dead bees at their entrances, especially the closest hive which was upwind and 1.1 km away (Euclidian distance), indicating that the phenomenon was geographically confined to where the aerial treatments had been conducted. Immediate discussions with the community found nobody who had conducted any private spraying, thereby ruling out other potential sources of insecticide that may have affected the bees. A couple of days later, a bee attractancy study was conducted using hydrogels and the sugar water mixture, both without fipronil. This study confirmed results of a prior investigation on Norfolk Island,^25^ as well as two other studies elsewhere in the world,^23^, ^24^ that bees are not attracted to hydrogels or to the liquid sugar mixture, thereby ruling out the possibility that bees fed from the bait. Another few days later visual inspection results from the Australian Department of Agriculture also confirmed that there were no mites or pathogens visible on the bee samples, nor were there any associated deformities on any bees, ruling out parasites and visibly discernible disease as a potential cause.

Three weeks after the first bee deaths were found, the presence of fipronil was confirmed by NMI with the three samples containing 0.025, 0.11 and 0.25 mg kg^–1^, respectively. Notably, the median lethal dose (LD_50_) of fipronil for bees is <5 ng per bee which is the equivalent of <0.05 mg kg^–1^.^26^ At this point all of the bee hives were opened, and it was determined from the absence of queens, lack of brood and depleted bee numbers, that 13 of the 14 hives were beyond survival. Therefore, the issue was not just a temporary impact. At this time one pollen sample, three handful‐sized honey samples and two handful‐sized honey comb samples were collected randomly from among four of the affected hives and also sent to NMI for GC–MS analysis for fipronil using the same method described previously. None of these samples were found to contain fipronil.

What still remained unclear was how the bees were contaminated with fipronil given that the fipronil essentially was secured within the hydrogels. Although there were theoretically possible options of bees coming into contact with the fipronil directly (i.e. by feeding or accidentally touching hydrogels), or indirectly by touching something else that had contacted the hydrogels (e.g. flowers), these options did not seem realistic given the sheer number of bees that died and the total volume of fipronil exposure that the hives must have obtained to result in complete collapse. To rapidly gain nonquantitative insight into the possibility that somehow the fipronil had been dispersed like an aerosol, we observed and described Argentine ant population levels at varying distances downwind from the drone flight paths. Because the aerial work treated only the central core of the infestation, theoretically only ants in and very close to the central core should display treatment effects. However, if an aerosol also had been present, then ants downwind and well into the untreated area would display treatment effects, too. Argentine ants had been very abundant and easy to find when the infestation was mapped before the commencement of baiting, and they should still have been very abundant in these untreated areas inspected at this time, two months after the incident. Instead, Argentine ants were very difficult to find close (≈20 m) to where the hydrogels were dropped, were present but in low numbers up to ≈100 m away from the drop zone, but remained extremely abundant and at abundance levels typically found in untreated areas at distances >100 m away from the drop zone. The gradient of ant activity was considered consistent with what would be expected if fipronil landed downwind of the drop zone in aerosol form.

#### 2.2.1 Study 1: fipronil in aerosol form

The first study simply aimed to determine if fipronil was indeed being released as an aerosol during aerial delivery of the bait. If so, samples collected downwind of the drone would contain fipronil, but those upwind would not. A large open area with a constant steady breeze, that also was bee‐free, was selected. Temperature was 20° C and the wind was 16 knots (5 m s^–1^) easterly, almost identical to the conditions when the baiting operation associated with the bee deaths was conducted.

**Figure 4:**
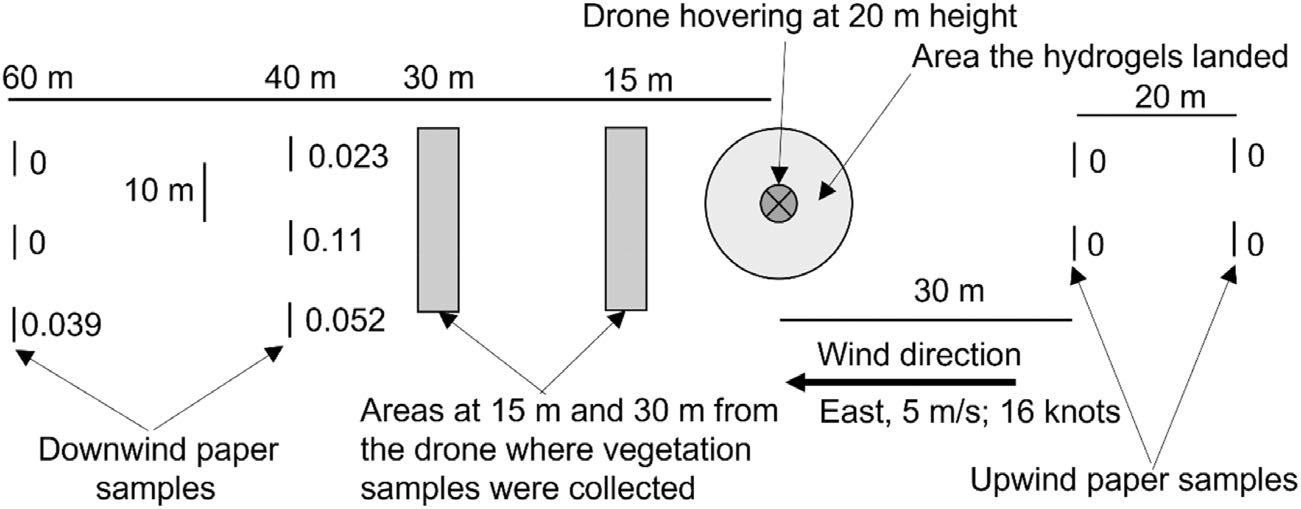
Design of Study 1 (NB not drawn to scale). Numbers beside the paper sample points are the fipronil concentrations obtained (mg kg^–1^).

The experimental design is shown in Fig. 4. Upwind and downwind of where the drone would drop a load of hydrogel ant bait, ten A4 pieces of paper were pinned to stakes at numerous distances, with their broad sides facing the drone to be exposed to any potential aerosols. The drone hovered at ≈20 m height, and dropped a full load of hydrogel ant bait (30 kg) during a period of ≈2 min. At the end of the flight the paper was collected and placed in individual plastic sleeves, with care being taken to not accidentally contaminate any of the samples with fipronil. One piece of paper not associated with this field trial also was placed in individual plastic sleeves to serve as a control, and a single hydrogel also was packaged so that the exact concentration of fipronil in that batch of bait could be quantified. Five days later, two vegetation samples also were opportunistically collected 15 and 30 m downwind of where the drone hovered in an extra attempt to potentially collect fipronil in the environment and also to gain some insight into fipronil persistence. The paper, hydrogel and vegetation samples were then sent to NMI for GC–MS quantification of fipronil presence.

#### 2.2.2 Study 2: dispersal of the aerosol

**Figure 5:**
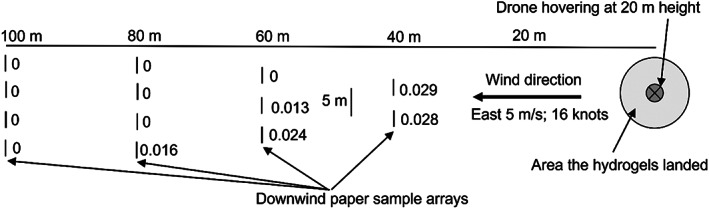
Design of Study 2 (NB not drawn to scale). Numbers beside the paper sample points are the fipronil concentrations obtained (mg kg^–1^).

This study was conducted after the results of Study 1 were obtained, and aimed to better quantify the dispersal distance and pattern of aerosols generated from aerial hydrogel dispersal, as well as to test whether free‐falling hydrogels would eliminate the production of aerosols. The study design was similar to that of Study 1, except that this time paper arrays were positioned solely downwind of the drone, with a focus to determine the far end of aerosol dispersal distances (Fig. 5). Based on results from Study 1 it was anticipated that 80 m would be the farthest distance fipronil would be collected with the drone operating at 20 m height. The same location, with near identical wind conditions, was used as in Study 1. Thirteen pieces of A4 paper were arranged as per Fig. 5. When all was prepared, and the drone was in position, the drone released a full load (30 kg) of hydrogels containing fipronil. Upon completion the paper samples were carefully collected as in Study 1. The experiment then was repeated in the same location without the use of the spinner such that the hydrogels free‐fell from the drone. However, this time only four paper samples were collected at the distance of 10 m based on the expectation that any potential aerosols from free‐falling hydrogels would need to be collected much closer than those generated by propulsion. All samples were sent to NMI for GC–MS quantification of fipronil presence.

#### 2.2.3 Study 3: first field trial with modified protocols

**Figure 6:**
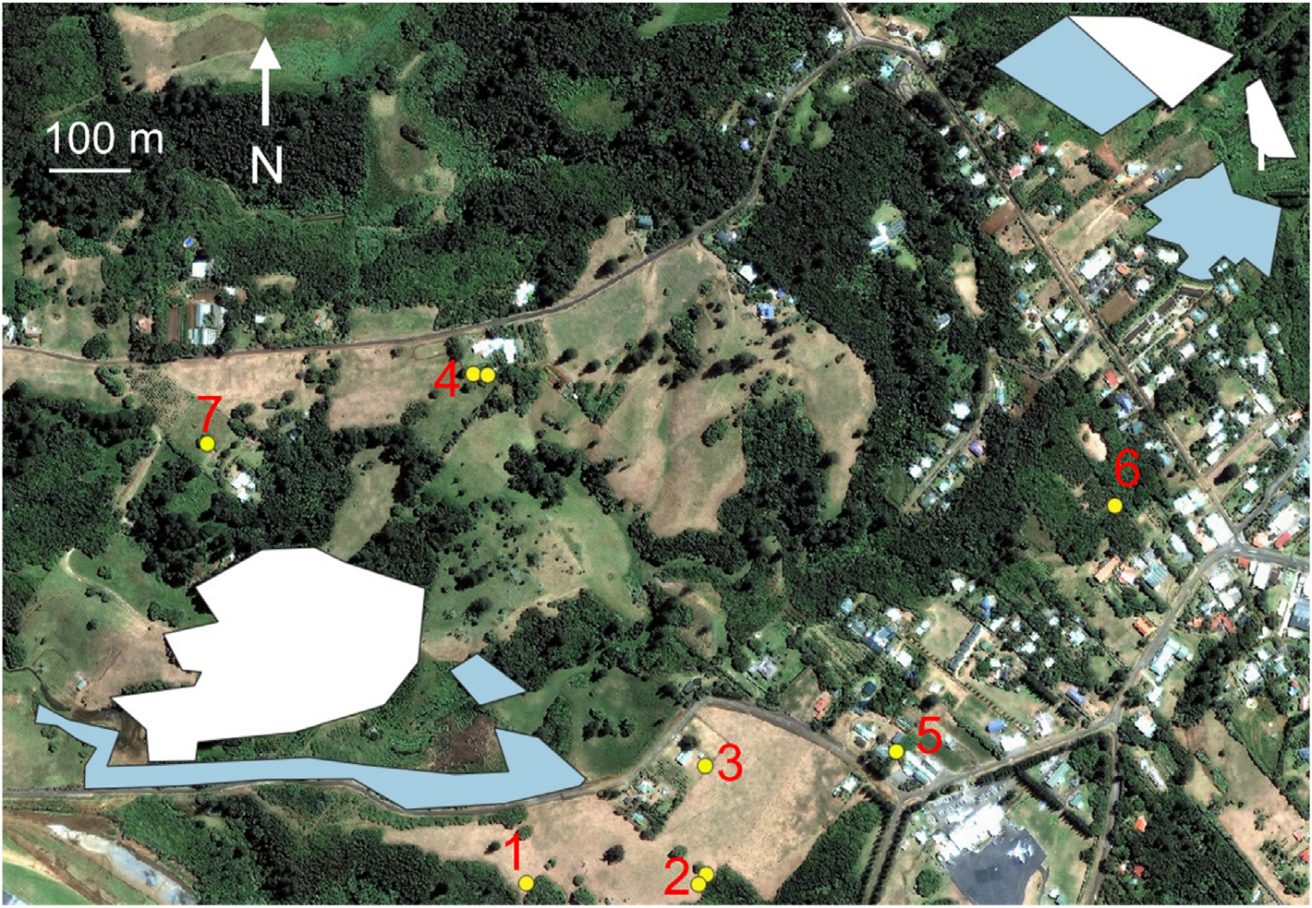
Map showing the areas to be treated aerially with hydrogels in Study 3, using the spinner (white polygon) and not using the spinner (blue polygons), as well as the numbered locations of bee hives within the local area (yellow points).

Study 3 involved the re‐commencement of broadscale aerial treatments over two Argentine ant populations (Fig. 6), and it was anticipated that bee hives >150 m distant would be safe from aerosols generated by the spinner, especially if they were not downwind of treatments. For this reason, the one hive that was known to be within the treatment area was moved, but all other bee hives located in the general area (all being >150 m from the treatment areas), were not moved but were visually monitored pre‐ and post‐treatment for dead bees at their entrances. The two ant populations were divided into areas that would be aerially treated with and without the spinner, based on potential for aerosol issues. Note that Argentine ants in the two populations living between these aerial treatment areas would be treated using ground‐based methods, but at later dates so that any potential bee issues could be unequivocally associated with aerial treatments only.

The NE population had three aerial treatment areas with a combined area of 6.1 ha, and there was only one hive within any close proximity, being 320 m away (Fig. 6). Between 19 January and 16 February 2022, 3000 kg of ant bait was dispersed over the treatment areas in seven treatments. Throughout this time the wind blew in all cardinal directions at strengths between 5 and 22 knots (2.8–11.4 m s^–1^). The SW population also had three aerial treatment areas covering 10.8 ha, with eight bee hives in six locations nearby. The most distant hive was 420 m away, and the existence of the hive at Location 1 >100 m from the treatment area was not known when treatments commenced. Two treatments were conducted over three days between 19 and 27 January 2021 with 1510 kg ant bait delivered. Bait application on the first treatment day covered all but a small section on the northern end of the large area, with prevailing southeasterly winds at 8 knots (4.2 m s^–1^). The remaining section was treated two days later with eastsoutheasterly winds blowing 13–17 knots (6.7–8.9 m s^–1^). Only seven dead bees were found at the entrance of one of the hives in location 2 on 25 January. The second treatment was conducted on 27 January over all three areas with easterly winds blowing at 13–22 knots (6.9–11.1 m s^–1^). A visual inspection of hives was conducted the following day, and 12 and 16 dead bees were found in hive locations 1 and 2, respectively. This time these hives were opened, and they were found to be queenless, and lacked eggs, indicating that the queens probably were killed after the first treatment. The dead bees were collected from the bee hives in these two locations and stored in a freezer. At this time, plans for additional treatments over the SW population ceased. Also at this time, none of the other surrounding hives had any dead bees at their entrances, but three days later one of the hives in location 4 was found to have 34 dead bees at the entrance, and when opened the queen and new eggs could not be found. These dead bees were collected, and were sent with the other bees collected before NMI for GC–MS assessment for the presence of fipronil. Monitoring continued at all hives until 3 May 2021.

#### 2.2.4 Study 4: subsequent field trial

This study involved broadscale treatments over 65 ha without the use of the spinner, and with all bee hives moved to distances >500 m from the treatment area. For 6 months before treatments, every landholder within and around the treatment area was contacted about the intent to conduct ant treatments and the need to move bee hives before the treatments commencing. Treatments commenced on 24 October 2021 and continued until 10 December 2021, with 26 970 kg of hydrogel bait delivered over the treatment area in six treatment rounds. Apiarists were requested to self‐monitor their bee hives and report any findings of concern.

## 3 RESULTS

### 3.1 Study 1: fipronil in aerosol form

**Figure 7:**
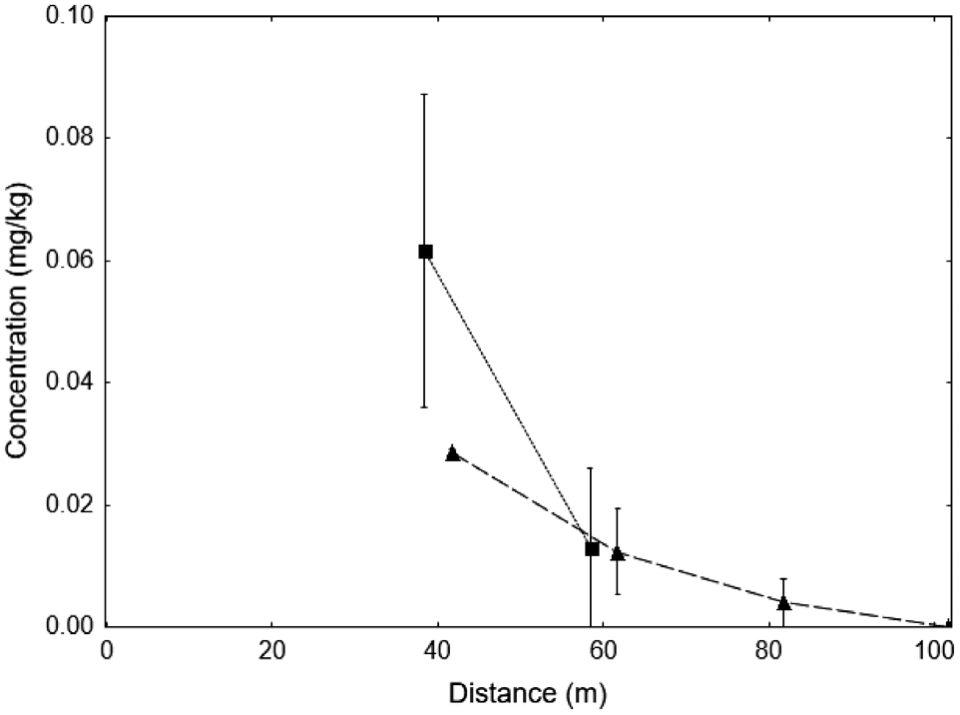
Mean (±SE) fipronil concentrations found on A4 sample sheets at set distances from a geostationary drone aerially dropping a load of hydrogel ant bait in Study 1 (squares) and Study 2 (triangles).

The results confirmed the hypothesis that fipronil was being dispersed as an aerosol during aerial baiting. The three closest downwind samples all had fipronil contamination, but only one of the farthest downwind sample had fipronil contamination (Fig. 4). The volume of fipronil captured declined with distance away from the drop zone (Fig. 7). None of the upwind samples had fipronil, nor did the control. Fipronil was not detected in any of the vegetation samples, but it is not clear if that is because no fipronil landed this close to the drop zone, or because any fipronil already had degraded when these samples were collected five days after the trial was conducted. The hydrogel contained 0.5 mg kg^–1^ fipronil, which is approximately one tenth the concentration that would have been used in the aerial baiting associated with the incident (6 mg kg^–1^). This low concentration was attributed to the difficulty of adding the correct minute fipronil amount to the very small test batch relative to the batches made for the ant treatments (30 *versus* 500 kg).

### 3.2 Study 2: dispersal of the aerosol

The results conformed to expectations with fipronil volume captured decreasing with increasing distance from the drone (Fig. 7). The pattern of zero captures (Fig. 5) probably indicates that there was some wind shear at the time of bait release, with the drift not travelling exactly perpendicular to the array. This potentially gave an underestimation of distance the aerosol travelled. When the trial was repeated without the use of the spinner, no fipronil was collected on any of the paper samples, or the volume was sufficinetly low that the NCI analysis could not detect it (<0.01 mg kg^–1^).

### 3.3 Study 3: first field trial with modified protocols

Toxicology results confirmed the presence of fipronil on the dead bees with the two samples returning concentrations of 0.16 and 0.1 mg kg^–1^. By 3 May (104 days after the first treatment was conducted) it was found that almost all hives in the area had numerous dead bees at their entrances, and therefore were affected by the treatments. Hive mortality (lack of queen and brood) was recorded in locations 5 and 6 in inspections conducted on 10 March and 1 April respectively. On 3May, the hive in Location 7 also was declared to have collapsed after 2 months of slow decline. Only one other hive was reported as having died during the same period throughout the rest of the island, with that death being attributed to prevailing environmental conditions, not the aerial treatments. Only the hive in Location 3 was seemingly not affected by the treatments, and the hive at Location 1 recovered from its initial decline.

### 3.4 Study 4 subsequent field trial

No reports of potential bee issues associated with the aerial baiting were received from the numerous apiarists with hives surrounding the treatment area.

## 4 DISCUSSION

The results of all of the studies fully support the theory that propelling moist hydrogels with the spinner‐created aerosols that were not readily observed visually. Study 1 confirmed the existence of aerosols, with fipronil detected on downwind samples. Study 2 quantified that in those specific wind conditions the aerosols were falling to the ground, with the maximum distance being at ≈30° from the ground to the dispersal point. Study 3 also found that free‐falling hydrogels either do not produce aerosols or, if so, at volumes too negligible to be collected or quantified. It confirmed that even bee hives upwind and many hundreds of metres away from the area being baited can be contaminated by the aerosols. Study 4 found that free‐falling hydrogels as well as moving bee hives >500 m from the treatment area resulted in no observed bee deaths or bee hive mortality.

What remains unclear is the exact mode of contamination. Fipronil is not systemic, so it is unlikely that it was taken up by plants and expressed in nectar. Likewise there was no standing water near any of the treatment areas, so fipronil was not able to contaminate water sources that bees may have utilized. One option is that foraging bees flew through the aerosols thereby directly contacting the fipronil. A second option is that bees are foraging on substrates such as flowers that the aerosols have landed on, thereby contacting the fipronil as they walk on the substrate. However, it is likely that both modes may have occurred depending upon local circumstances. It is notable that fipronil is well‐known to be highly effective as a contact insecticide,^27^, ^28^, ^29^, ^30^, ^31^ so the bees are not necessarily consuming it in the field, but would certainly be affected when they groom themselves, groom others or move dying nestmates out of the hive. If the contamination is being sourced from the substrates, it also appears that this risk does not persist long. Potential evidence from Study 1 is that no fipronil was detected on the vegetation samples collected 15 and 30 m from the bait drop area five days after the bait application. However, it also is potentially possible that no aerosols fell at these distances. Stronger evidence for short environmental persistence is from the apiarist affected by the original incident replacing and restocking most of the bee hives 29 days after the second treatment, and those hives thrived.

Bees are capable of flying multiple kilometres to forage for nectar,^32^ with such distances flown being determined in part by prevailing environmental conditions,^33^ and the quality and quantity of available resources.^34^ So, potentially, foragers from hives far from the treatment area could have been affected, which in turn could have impacted those in distant hives. At least for entire hives this does not appear to have occurred in the treatments presented here, with hives located in all other locations on the island not being found or reported as having been impacted by the treatments when the spinner was being used. Whether some foragers from farther hives died before returning to the hives, or so few returned contaminated that the impact was negligible, remains unknown.

Two other details remain unclear. First, why in the original incident bees were not found affected after the first treatment, rather than the second treatment which was applied a week later. Nothing was greatly different about the environmental conditions of those two treatments. Second, why a bee hive within location 2 of Study 3 apparently was not affected in 2018 when extensive aerial treatments were applied eight times right up to beside that hive. Clearly, though, the impact of the inadvertent production of aerosols resulting from propelled dispersal of hydrogels was always going to be at its most evident when treatments were conducted within close proximity to bee hives. Had that hive died or been found to have excessive bee death during those 2018 treatments, perhaps this effect may have resulted in a much smaller incident.

The aerosol issue is likely to occur with every motorized mechanism dispersing hydrogels. However, although much of what has been presented here should serve as general guidance for potential future baiting using propulsion apparatus, it should not be expected that the quantifications of the aerosol issue found here will be consistent using other equipment, hydrogel forms or in other conditions. Most likely the volume and physical size of the aerosol particles would differ according to the propulsion mechanism (i.e. spinner size and speed, air propulsion), the numerous types of hydrogels available, as well as the different viscosities of the liquids they could absorb, for example resulting from varying sugar concentrations. Also, the aerosol dispersal distance and area will be determined by the aerosol particle size, wind speed and drop height. Essentially what has been presented here regarding the dispersal of the aerosol is specific to these conditions. Notably, we doubt that just reducing the concentration of the active constituent (fipronil) would reduce or eliminate the issue, especially given that it was used here in very minute concentrations (equivalent to 0.006 g kg^–1^). Changing the active constituent to a different general insecticide also would not be likely to eliminate the risk of such nontarget impacts.

The work presented here relates to aerosols created from moist ant bait, yet it is possible that the same issue is happening with solid dry products if they produce a fine dust when dispersed. Such dust would have just as many implications as aerosols for the inadvertent dispersal of chemicals, especially for organic properties, areas containing farm animals or produce, rainwater harvesting from roof tops, open bodies of water, for direct exposure of other nontarget species, as well as people. Further research into this issue is warranted given the potential for social, environmental and economic impacts of ant eradication programs globally.

## CONFLICT OF INTEREST

All authors declare that they have no conflicts of interest.

## Data Availability

The data that support the findings of this study are available from the corresponding author upon reasonable request.

## References

[1] Howald G , Donlan CJ , Galván JP , Russell JC , Parkes J , Samaniego A *et al*., Invasive rodent eradication on islands. Conserv Biol 21:1258–1268 (2007). 17883491 10.1111/j.1523-1739.2007.00755.x

[2] Wilkinson IS and Priddel D , Rodent eradication on Lord Howe Island: challenges posed by people, livestock, and threatened endemics, in Island Invasives: Eradication and Management, ed. by Veitch CR , Clout MN and Towns DR . IUCN, Gland, Switzerland, pp. 508–514 (2011).

[3] Thomas B , Taylor R , Dunlevy P , Mouritsen K and Kemp J , The Ka mate reverse‐bait snap trap – a promising new development, in Island Invasives: Eradication and Management, ed. by Veitch CR , Clout MN and Towns DR . IUCN, Gland, Switzerland, pp. 233–238 (2011).

[4] Blackie HM , MacKay JW , Allen WJ , Smith DH , Barrett B , Whyte BI *et al*., Innovative developments for long‐term mammalian pest control. Pest Manag Sci 70:345–351 (2014). 23943626 10.1002/ps.3627

[5] Gaigher R , Samways MJ , Joliffe KG and Jolliffe S , Precision control of ant invasive ant on an ecologically sensitive tropical Island: a principle with wide applicability. Ecol Appl 22:1405–1412 (2012). 22908700 10.1890/11-2002.1

[6] Buczkowski G , The Trojan horse approach for managing invasive ants: a study with Asian needle ants, *Pachycondyla chinensis* . Biol Invasions 18:507–515 (2016).

[7] Green P , Comport S , Slip D , The Management and Control of the Invasive Alien Crazy Ant (*Anoplolepis Gracilipes*) on Christmas Island, Indian Ocean: The Aerial Baiting Campaign September 2002. Report to Environment Australia and the Crazy Ant Steering Committee. Monash University, Melbourne (2004).

[8] Howald G , Donlan CJ , Faulkner KR , Ortega S , Gellerman H , Croll DA *et al*., Eradication of black rats *Rattus rattus* from Anacapa Island. Oryx 44:30–40 (2009).

[9] Board LHI , Lord Howe Island Rodent Eradication Project – Public Environment Report. Lord Howe Island Board, Lord Howe Island (2016).

[10] Williams DF , Collins HL and Oi DH , The red imported fire ant (hymenoptera: Formicidae): an historical perspective of treatment programs and the development of chemical baits for control. Am Entomol 47:146–159 (2001).

[11] Klotz JH , Rust MK , Costa H , Reierson DA and Kido K , Strategies for controlling argentine ants (hymenoptera: Formicidae) with sprays and baits. J Agr Urban Entomol 19:85–94 (2002).

[12] Hoffmann BD , Abbott KL and Davis P , Invasive ant management, in Ant Ecology, ed. by Lach L , Parr CL and Abbott KL . Oxford University Press, Oxford, pp. 287–304 (2010).

[13] Tekin B and Sindir O , Variable rate control system designed for spinner disc Fertiliser spreader–“pre Fer”. Agric Eng 2:45–53 (2013).

[14] Balafoutis A , Beck B , Fountas S , Vangeyte J , Van der Wal T , Soto I *et al*., Precision agriculture technologies positively contributing to GHG emissions mitigation, farm productivity and economics. Sustainability 9:1339 (2017).

[15] Broome KG , Golding C , Brown KP , Horn, S , Corson P , and Bell P , Mouse eradication using aerial baiting: current agreed best practice used in New Zealand (version 1.0). New Zealand Department of Conservation Internal Document DOC‐3034281, Wellington, New Zealand (2017).

[16] Boser CL , Hanna C , Faulkner KR , Cory C , Randall JM and Morrison SA , Argentine ant management in conservation areas: results of a pilot study. Monogr West N Am Nat 7:518–530 (2014).

[17] Cabrera ME , Fontan IR , Hoffmann BD and Josens R , Laboratory and field insights into the dynamics and behavior of the argentine ant *Linepithema humile* feeding from hydrogels. Pest Manag Sci 77:3250–3258 (2021). 33729652 10.1002/ps.6368

[18] Buczkowski G , Roper E and Chin D , Polyacrylamide hydrogels: an effective tool for delivering liquid baits to pest ants (hymenoptera: Formicidae). J Econ Entomol 107:748–757 (2014a). 24772557 10.1603/ec13508

[19] Buczkowski G , Roper E , Chin D , Mothapo N and Wossler T , Hydrogel baits with low‐dose thiamethoxam for sustainable argentine ant management in commercial orchards. Entomol Exp Appl 153:183–190 (2014b).

[20] Peck RW , Banko PC , Donmoyer K , Scheiner K , Karimi RR and Kropidlowski S , Efforts to eradicate yellow crazy ants on Johnston atoll: results from crazy ant strike teams X, XI and XII (June 2015 December 2016). CTIT Tech Rep Ser (2017).

[21] Buczkowski G , Hydrogel baits pose minimal risk to non‐target insects and beneficial species. Entomol Exp Appl 168:948–955 (2020).

[22] Krushelnycky P , Evaluation of water‐storing granules as a promising new baiting tool for the control of invasive ants in Hawaii, in Final Report to the Hawaii Invasive Species Council Covering FY18 and FY19 Project Funding. University of Hawaii at Manoa, Hawaii (2021).

[23] Krushelnycky PD and Gillespie RG , Sampling across space and time to validate natural experiments: an example with ant invasions in Hawaii. Biol Invasions 12:643–655 (2010).

[24] Menke S , Ward P and Holway D , Long‐term record of argentine ant invasions reveals enduring ecological impacts. Ecology 99:1194–1202 (2018). 29504667 10.1002/ecy.2200

[25] Hoffmann BD , Honey bees are not attracted to multiple new ant baits containing sugar. B. Entomol Res (in press). 10.1017/S0007485322000451 36111521

[26] Edwards ED , Woolly EF , McLellan RM and Keyzers RA , Non‐detection of honeybee hive contamination following Vespula wasp baiting with protein containing fipronil. PLoS One 13:e0206385 (2018). 30372501 10.1371/journal.pone.0206385 PMC6205613

[27] Soeprono AM and Rust MK , Effect of horizontal transfer of barrier insecticides to control argentine ants (hymenoptera: Formicidae). J Econ Entomol 97:1675–1681 (2004a). 15568359 10.1603/0022-0493-97.5.1675

[28] Soeprono AM and Rust MK , Effect of delayed toxicity of chemical barriers to control argentine ants (hymenoptera: Formicidae). J Econ Entomol 97:2021–2028 (2004b). 15666760 10.1093/jee/97.6.2021

[29] Choe DH and Rust MK , Horizontal transfer of insecticides in laboratory colonies of the argentine ant (hymenoptera: Formicidae). J Econ Entomol 101:1397–1405 (2008). 18767753 10.1603/0022-0493(2008)101[1397:htoiil]2.0.co;2

[30] Buczkowski G , Trap‐treat‐release: horizontal transfer of fipronil in field colonies of the black carpenter ant, *Camponotus pannsylvanicus* . Pest Manage Sci 75:2195–2201 (2019). 10.1002/ps.5345 30663198

[31] Buczkowski G and Wossler TC , Controlling invasive argentine ants, *Linepithema humile*, in conservation areas using horizontal insecticide transfer. Sci Rep 9:19495 (2019). 31863081 10.1038/s41598-019-56189-1 PMC6925143

[32] Beekman LM and Ratnieks LW , Long‐range foraging by the honey‐bee, Apis mellifera. Funct Ecol 14:490–496 (2000).

[33] Steffan‐Dewenter I and Kuhn A , Honeybee foraging in differentially structured landscapes. Proc R Soc Lond Ser B Biol Sci 270:569–575 (2003). 10.1098/rspb.2002.2292 PMC1691282 12769455

[34] Gary NE , Witherell PC and Marston J , Foraging range and distribution of honey bees used for carrot and onion pollination. Environ Entomol 1:71–78 (1972).

